# Protocol for understanding acute sarcopenia: a cohort study to characterise changes in muscle quantity and physical function in older adults following hospitalisation

**DOI:** 10.1186/s12877-020-01626-4

**Published:** 2020-07-10

**Authors:** Carly Welch, Carolyn A. Greig, Tahir Masud, Thomas Pinkney, Thomas A. Jackson

**Affiliations:** 1 Medical Research Council and Versus Arthritis Centre for Musculoskeletal Ageing Research, University of Birmingham and University of Nottingham, Birmingham and Nottingham, UK; 2 Institute of Inflammation and Ageing, University of Birmingham, Birmingham, UK; 3 University Hospitals Birmingham NHS Foundation Trust, Birmingham, UK; 4 University of Birmingham Research Laboratories, Queen Elizabeth Hospital Birmingham, Mindelsohn Way, Edgbaston, Birmingham, B152GW UK; 5 Birmingham Biomedical Research Centre, University of Birmingham and University Hospitals Birmingham NHS Foundation Trust, Birmingham, UK; 6 School of Sport and Exercise Sciences, University of Birmingham, Birmingham, UK; 7 School of Sport, Exercise and Rehabilitation Sciences, University of Birmingham, Edgbaston, Birmingham, B15 2TT UK; 8 University of Nottingham, Nottingham, UK; 9 Nottingham University Hospitals NHS Trust, Nottingham, UK; 10 Clinical Gerontology Research Unit (CGRU), First Floor, South Corridor, City Hospital, Nottingham, NG5 1PB UK; 11 Academic Department of Surgery, University of Birmingham, Room 29, 4th Floor, Heritage Building, Edgbaston, Birmingham, B15 2TH UK; 12 University of Birmingham Research Laboratories, Queen Elizabeth Hospital Birmingham, Mindelsohn Way, Edgbaston, Birmingham, B152GW UK

**Keywords:** Acute sarcopenia, Physical function, Older adults, Hospitalisation

## Abstract

**Background:**

Older adults are vulnerable to the effects of acute sarcopenia (acute muscle insufficiency) following hospitalisation. However, this condition remains poorly characterised to date. It is hypothesised that acute sarcopenia arises due to a combination of bed rest and inflammatory surge. This study aims to characterise changes in muscle quantity and function, determining which factors (clinical and biological) are most predictive, and how these relate to change in physical function at 13 weeks.

**Methods:**

This study will include three groups of patients aged 70 years and older; patients undergoing elective colorectal surgery, patients admitted for emergency abdominal surgery, and patients admitted under general medicine with acute bacterial infections. Changes in muscle quantity (Bilateral Anterior Thigh Thickness with ultrasound and bioelectrical impedance analysis) and muscle function (muscle strength, physical performance) within 1 week of hospitalisation or surgery will be characterised, with follow-up of patients at 13 weeks. Physical function will be measured using the Patient Reported Outcome Measures Information System, and the Short Physical Performance Battery (or gait speed alone within 1 week of surgery).

**Discussion:**

This study will fully characterise changes in muscle quantity and function in hospitalised older adults and enable risk stratification towards targeted interventions in clinical practice. The results of this study will inform further research involving interventions to ameliorate changes.

**Trial registration:**

ClinicalTrials.gov Identifier: NCT03858192; Prospectively registered 28th February 2019.

## Background

Acute sarcopenia is an emerging condition of acute muscle insufficiency; older adults are considered particularly vulnerable to its effects following hospitalisation [1]. The European Working Group on Sarcopenia in Older People 2 (EWGSOP2) defines sarcopenia as reduced muscle strength with reduced muscle quantity or quality; cut-off values to meet criteria are usually set 2.0–2.5 standard deviations below the mean of a young adult healthy reference population. Additional demonstration of low physical performance is defined as severe sarcopenia. The revised definition (EWGSOP2) includes a distinction between acute and chronic sarcopenia; acute sarcopenia is defined as incident sarcopenia within 6 months, normally following a stressor event [2]. However, acute sarcopenia has been poorly characterised to date [1].

The biological mechanisms, clinical risk factors, longer term outcomes, and most effective management strategies of acute sarcopenia are currently unknown. Acute sarcopenia is considered to be caused by a combination of heightened inflammation and muscle disuse during bedrest. Studies involving healthy volunteers have demonstrated that bedrest is associated with declines in muscle quantity, strength, and aerobic performance, and that this effect is exacerbated by age [3, 4]. Acute illness (e.g. acute bacterial infection) and major surgery are associated with systemic inflammatory response [5] and endocrinological stress response (e.g. increased cortisol, decreased dehydroepiandrosterone sulfate (DHEA-s)) [6]. Pro-inflammatory cytokines activate pathways leading to increased muscle protein degradation [7] and hypercortisolaemia has been shown to exacerbate loss of muscle quantity during bedrest [8]. It has been postulated that acute sarcopenia may be partially recoverable, but may increase the risk of chronic sarcopenia over time [1]. It is proposed to be related to a combination of acute inflammatory surge and bedrest during hospitalisation [1]. Characterising acute sarcopenia will enable greater understanding of the significance of changes in clinical practice, and allow risk stratification towards targeted interventions.

EWGSOP traditionally recommended Computed Tomography (CT), Magnetic Resonance Imaging, or Dual-Energy X-Ray Absorptiometry to measure muscle quantity [9]. However, these tests cannot be used at the bedside and have limitations when used serially [10]. Ultrasound measurement of Bilateral Anterior Thigh Thickness (BATT) has excellent inter-rater and intra-rater variability [11]. EWGSOP2 supports use of ultrasound for clinical assessment of sarcopenia [2] and a consensus protocol has been proposed [12]. Bioelectrical Impedance Analysis (BIA) is an alternative non-invasive tool that provides estimates of lean mass. Muscle quantity measured by BIA has been shown to correlate with BATT [13], however, BIA is more greatly affected by fluid balance [14, 15]. BIA is also not currently recommended for use on people with implantable cardiac devices, although research suggests this is likely to be safe [16].

Colorectal surgery is commonly performed on older adults [17]. It is not typically associated with cachexia, when performed for localised colorectal cancer [18]; metastatic cancer is known to be associated with increased risk of cachexia compared to localised cancer [19]. Colorectal surgery patients do not typically present with disease-associated pre-operative functional decline associated [20], as compared to orthopaedic or vascular surgery, where impairments in function are presenting symptoms of the illnesses themselves [21, 22]. This offers the opportunity for pre-insult measurements to be taken prior to hospitalisation. Previous studies have demonstrated acute declines in handgrip strength and muscle quantity using BIA in older adults admitted electively for colorectal surgery [23]. Acute reductions in BATT and usual gait speed were also demonstrated in our pilot study, which was used to refine this protocol [24]. Interestingly, an apparent increase in BATT was demonstrated immediately postoperatively [24]; this may be related to fluid balance but warrants further investigation [25]. However, emergency admitted patients may be at the greatest risk of declines in muscle quantity and function due to increased inflammation. Within the UK, hospitalised older adults are most commonly admitted to general medicine wards [26]. Studies involving medical and orthopaedic patients have shown variable changes in muscle quantity and function in hospitalised older adults [23, 27, 28], and changes have not been evaluated in patients admitted for emergency abdominal surgery.

## Methods

### Aim

To clinically and biologically characterise acute sarcopenia in older hospital populations, assessing for within group differences in elective colorectal surgery, emergency surgery, and general medicine patients. This will enable determination of mechanisms and identification of potential intervention strategies.

### Design and setting

**Fig. 1:**
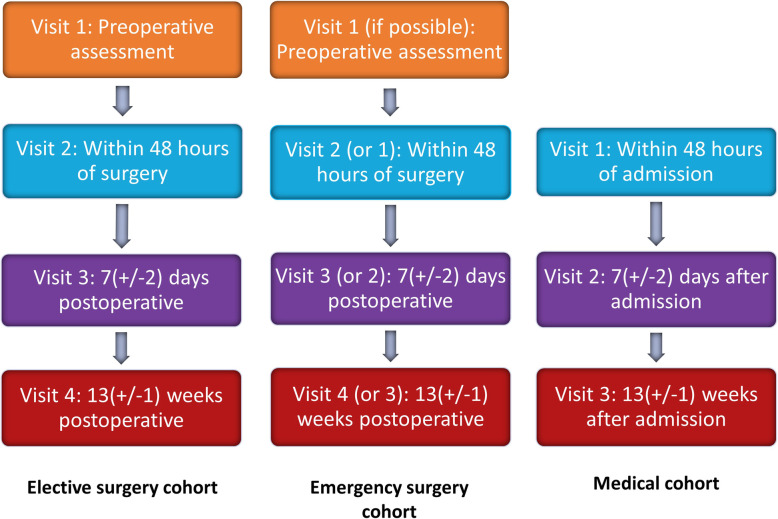
Study schema for recruitment and follow-up of each included cohort

This is a single site cohort study at the Queen Elizabeth Hospital Birmingham (QEHB), involving 56 elective colorectal, 56 emergency abdominal surgery, and 56 medical patients. QEHB is a large tertiary hospital, with a firmly embedded research infrastructure. In the elective cohort, measurements will be performed in preoperative assessment clinic, within 48 h of surgery, at 7 days postoperatively (+/− 2 days), and at 13 weeks postoperatively (+/− 1 week). In the emergency surgery cohort, we aim to recruit participants preoperatively where possible. Where this is not possible, we will recruit participants within 48 h of emergency surgery; further assessments will be performed at 7 days postoperatively (+/− 2 days), and at 13 weeks postoperatively (+/− 1 week). Medical patients will be recruited within 48 h of admission with further assessment at 7 days post-admission (+/− 2 days), and at 13 weeks post-admission (+/− 1 week). The timeframe of 13 weeks has been chosen pragmatically as a timeframe that was considered important to our patient and public involvement panel that could be feasibly conducted without high drop-out rates. The full study schema is shown in Fig. 1.

### Characteristics of participants

The elective cohort will include patients expected to undergo major colorectal surgery, the emergency surgery cohort will include emergency admitted patients who have undergone or are planned to undergo emergency abdominal surgery, and the medical cohort will include emergency admitted patients with (or suspected) acute bacterial infections. Participants aged 70 years or older at time of recruitment will be included in all cohorts. Participants who are unable to provide written informed consent at time of recruitment will not be included in the elective cohort, although specific consent will be obtained for participants to remain in the study if they lose capacity, including details of any named consultee. In the emergency surgery and medical cohorts, personal or professional consultee declaration will be obtained if the participant is unable to provide written informed consent. Participants who are unable to understand verbal English, who were unable to mobilise prior to admission to hospital, or who have a life expectancy of less than 30 days will be excluded from all cohorts.

### Processes and interventions

Table 1 shows the complete schedule of assessments that will be performed during this study. We describe the procedures that will be performed at each visit in further detail below.

**Table 1:** Schedule of study procedures. This chart shows all possible visits for each cohort and assessments that would be expected to take place for each participant in each cohort. Visits marked with * may not take place for all participants

Visit	Elective cohort				Emergency cohort				Medical cohort		
	A	B	C	D	A*	B	C	D	B	C	D
**Demographics, observations, medications, medical history, blood tests as part of routine care**	**✓**	**✓**	**✓**		**✓**	**✓**	**✓**		**✓**	**✓**	**✓**
**Review of CT scans performed as part of routine clinical care (if available)**	**✓**				**✓**						
**BATT using ultrasound**	**✓**	**✓**	**✓**	**✓**	**✓**	**✓**	**✓**	**✓**	**✓**	**✓**	**✓**
**BIA**	**✓**	**✓**	**✓**	**✓**	**✓**	**✓**	**✓**	**✓**	**✓**	**✓**	**✓**
**Handgrip strength**	**✓**	**✓**	**✓**	**✓**	**✓**	**✓**	**✓**	**✓**	**✓**	**✓**	**✓**
**Short Physical Performance Battery**	**✓**			**✓**				**✓**	**✓**	**✓**	**✓**
**Gait speed alone**			**✓**				**✓**				
**Physical function by PROMIS**	**✓**		**✓**	**✓**	**✓**		**✓**	**✓**	**✓**	**✓**	**✓**
**Katz ADLs and Lawton IADLs**	**✓**		**✓**	**✓**	**✓**		**✓**	**✓**	**✓**	**✓**	**✓**
**Mini Nutritional Assessment (Full)**	**✓**			**✓**	**✓**			**✓**	**✓**		**✓**
**Extra frailty assessments**	**✓**		**✓**	**✓**	**✓**		**✓**	**✓**	**✓**	**✓**	**✓**
**Venepuncture (optional)**	**✓**	**✓**			**✓**	**✓**			**✓**		
**Application of physical activity recorder (optional)**		**✓**				**✓**			**✓**		
**Delirium assessment**		**✓**	**✓**		**✓**	**✓**	**✓**		**✓**	**✓**	
**Fluid balance assessment**		**✓**	**✓**		**✓**	**✓**	**✓**		**✓**	**✓**	
**Participant feedback**				**✓**				**✓**			**✓**

*PROMIS* Patient Reported Outcome Measures Information System, *ADLs* Activities of Daily Living.

Visit A: Preoperative assessment; in preoperative assessment clinic for elective cohort, on ward prior to surgery for emergency surgery cohort (where possible) – not applicable to medical cohort.

Visit B: Immediate; Within 48 h of surgery for surgical cohorts, within 48 h of admission for medical cohort.

Visit C: One week; 7 (+/− 2) days after surgery (surgical cohorts) or after admission (medical cohort).

Visit D: Three months; 13 (+/− 1) week after surgery (surgical cohorts) or admission (medical cohort).

### Muscle quantity assessment

#### Quadriceps ultrasound

Rectus Femoris (RF) and Vastus Intermedius (VI) muscles in both legs will be assessed using two-dimensional B-mode ultrasonography with a linear probe, as previously described [11]. This will be performed at first visit, immediately postoperatively (where applicable), at 7 day follow-up, and at 13 week follow-up. Participants will be positioned semi-upright with knees resting at 10–20° and advised to relax their muscles. The distance from greater trochanter to knee lateral joint line will be recorded and a mark placed on the skin mid-way between the two points. Measurements will be taken in line horizontally with these marks. Contact gel will be applied. Muscle thickness will be measured with the probe in transverse position. Depth will be adjusted until the femur and overlying structures are visible. The probe will be positioned such that the widest area of the RF appears over the midpoint of the femur. Frozen images at this location will be taken with the probe held in maximal relaxation.

Thickness measurements of subcutaneous tissues (SC), RF, and VI in a vertical line will be recorded, not including the fascia. Three frozen images will be used for all patients; a further image will be taken if there is greater than 10% variability between measurements. The mean of each reading will be used for analysis. BATT will be calculated as total thickness of right VI + right RF + left VI + left RF. BATT: SC ratio (BATT-SCR) will be calculated as BATT divided by total thickness of right SC + left SC [24]. Where possible, cross-sectional area of the right and left RF will be measured. All measurements will be performed by an investigator with training in taking these measurements. The reliability of BATT has been shown to be excellent when using the same protocol and same machine (intraclass coefficients > 0.9 for both intra-rater and inter-rater variability) [11].

A further image will be taken in the longitudinal position at each visit. Images will be saved and downloaded for assessment. RF and SC echogenicity will be determined using grey-scale analysis on Image J software. Pennation angle will be measured by the angle of insertion of the fascicles within the VI to the deep aponeurosis. The mean measure from up to three fascicles measured on each image will be used for analysis [29].

#### Bioelectrical impedance analysis (BIA)

BIA measurements will be taken at the first visit, immediately postoperatively (where applicable), at 7 day follow-up, and at 13 week follow-up using a multi-frequency analyser; Bodystat Quadscan 4000. This will not be performed if the participant has an implanted permanent pacemaker or defibrillator. The participant will be positioned lying semi-upright with knees resting in extension on the examination couch, hospital bed or equivalent. The assessor will ensure that limbs are not touching. Two electrodes will be placed on the right foot; one below the base of the toes and the other on the ankle between the medial and lateral malleoli. The red alligator clip will be attached to the electrode nearest the toes and the black to the one at the ankle. A further two electrodes will be placed on the right hand; one behind the knuckles and the other on the wrist next to the ulnar head. The red alligator clip will be attached to the electrode nearest the fingers and the black to the one at the wrist. Electrodes will be placed transversely so that the non-stick electrode connector is facing the researcher. The Bodystat Quadscan 4000 includes a quality control feature; an impedance graph is displayed prior to results being displayed. If the graph shows a smooth curve, the investigator will proceed to record results. If there are any bumps in the graph, the investigator will recheck lead and limb position prior to repeating the analysis. Single measurements will be recorded at each visit.

All returned measures including prediction marker, impedance, resistance, reactance, phase angle, fat weight, lean weight, dry lean weight, Fat Free Mass Index (FFMI), Body Fat Mass Index (BFMI), total body water, extracellular water, and intracellular water will be recorded. Skeletal muscle mass (SMM) will be additionally estimated using three previously validated equations: 1) SMM = 0.566 x Fat Free Mass (lean mass) [30]; 2) SMM = [((height^2^/ resistance) × 0.401) + (sex × 3.825) + (age x − 0.071)] + 5.102. In the second equation, height is in cm, for sex male = 1, female = 0, and age is in years [31]. 3) SMM = − 3.964 + (0.227 x (height^2^/ resistance)) + (0.095 x weight) + (1.384 x sex) + (0.064 x reactance) [32]. For all equations, the skeletal muscle index (SMI) will be calculated through the formula SMI = SMM/ height^2^_,_ where height is in m, for comparison with normative populations [33]. Height and weight are recorded for all patients at the site of this study as part of routine clinical care. For the elective cohort, height will be measured in preoperative assessment clinic using a stadiometer. For the emergency surgery and medical cohorts, height will be recorded using a stadiometer where possible. Where this is not possible, height will be taken from previous clinical records if these are available, or from patient report. If none of these methods are possible, then height may be estimated by measuring ulna length and conversion as per British Association of Parenteral and Enteral Nutrition guidelines [34]. If an estimate has been used, this will be recorded. The same height will be used for all visits.

#### L3-CT using imaging performed during routine medical care

CT scans will be reviewed if these have been performed as part of routine care and are available. Skeletal muscle index will be calculated at the level of the third lumbar vertebra (L3) on the first image with both vertebral spines visible using local hospital site Picture Archiving and Communication Software. This will be calculated by manually identifying skeletal muscles and automatic calculation of cross-sectional area; this value will be corrected for height^2^ [35]. Total psoas area (TPA) will also be calculated on the same slice. The right and left psoas muscle borders will be manually outlined and TPA will be calculated within the selected area. This measurement will be corrected for height^2^ [36]. This will be performed by the investigating geriatrician who is trained in use of the software.

### Muscle function assessment

#### Muscle strength

Handgrip strength will be measured at first visit, immediately postoperatively (where applicable), at 7 day follow-up, and at 13 week follow-up using a Jamar handheld dynamometer. Where the participant can sit in a chair, handgrip strength will be measured with the elbow flexed at 90^O^ and the forearm supinated. If measurements are taken in the bed this will be recorded; measurements will instead be performed in the most feasible upright position. Participants will be asked to “squeeze as hard as [they] can”. Handgrip strength will be measured twice on each side and the highest recording of the four measurements will be used for analysis [37].

#### Physical performance

The Short Physical Performance Battery (SPPB) is a standardised measure of physical performance that has been shown to be sensitive to change and provides an objective measure of physical function [38]. SPPB consists of usual gait speed, side-by-side stand, semi-tandem stand, tandem stand, and five chair stands (as quickly as they can). A total score of 12 is derived, with a lower score representing reduced physical performance. The SPPB will be measured at baseline and 3 month follow-up for the elective cohort, at 3 month follow-up for the emergency surgery cohort, and at all visits for the medical cohort. Gait speed alone will be measured at 7 day follow-up for both surgical cohorts. Gait speed will be measured by asking the participants to walk a four metre course at their “usual pace”. Gait speed will not be performed at recruitment in the emergency surgery cohort as this is considered unfeasible due to pain and immediate operative recovery. Measuring chair stands at 1 week post-operatively would cause increased abdominal strain, therefore, gait speed alone will be measured at this timepoint.

### Physical function – patient reported outcome measures information system (PROMIS®)

PROMIS physical function is a validated measure of physical function [39, 40]. PROMIS is an initiative that compares participant responses to a reference population and derives a T-score, where 50 is the mean, and 10 is the standard deviation. PROMIS will be measured at baseline and 3 month follow-up for all groups. The raw scores will be entered into the HealthMeasures scoring service, powered by Assessment Center^SM^ to derive T-scores.

### Comprehensive geriatric assessment

#### Demographics and comorbidities

Participant demographics, observations, medications, medical history, smoking and alcohol history, and blood test results performed as part of routine care will be collected. Medical comorbidities will be used to derive the Geriatric Index of Comorbidity [41]. Number of admissions and falls over the previous year will be recorded. Falls will be recorded from participant report. Any new information including changes in weight, observations, medications, or blood tests will be recorded at each visit, dependent on when these are recorded as part of usual clinical care.

#### Nutritional assessment

The Mini-Nutritional Assessment (MNA**®**) is a validated assessment tool for nutritional status [42]. Much of the information required will be collected elsewhere. Additional information that will be collected will include food intake, specifically protein and fruit or vegetable intake, and fluid intake from participant report. Mid-arm circumference will be measured at the mid-point between the olecranon and acromium. Calf circumference will be measured as the widest part of calf. These measurements will be taken for the dominant limb. The MNA (full form) will be assessed at baseline visits and 13 week follow-up visits for each group.

#### Frailty assessment

Frailty will be assessed at first visit, 7 day follow-up, and 13 week follow-up using the Frailty Index (FI) [43], 9-point Clinical Frailty Scale (CFS) [44], and phenotype definition [45]. Activities of Daily Living (ADLs) will be assessed using Katz (basic ADLs) [46] and Lawton (instrumental ADLs) [47] tools. The phenotypic diagnosis of frailty will be made if the participant meets three out of five criteria: low gait speed, low handgrip strength, weight loss, self-reported exhaustion or low physical activity. Cut-offs used in the original phenotype diagnosis will be used for gait speed, handgrip strength, and weight loss. Self-reported exhaustion will be defined if the participant answers “most of the time” or “all of the time” to how often over the last week they had felt that either “everything [they] did was an effort” or they “could not get going” [45]. Physical activity will be defined through self-report by asking the participant if over the last 3 months they have performed no weight-bearing physical activity, been for a short walk once/ month or less, or spent more than 4 hours/ day sitting [48]. The FI will be calculated by counting the total number of deficits present out of 36 defined criteria, and dividing by 36. These criteria have been adapted for secondary care use from those previously validated in a UK community setting to form the electronic frailty index (Supplementary File 1) [49]. The CFS will be determined by the investigating geriatrician after clinical review and after all other information has been collected. The investigating geriatrician will determine this immediately after reviewing the participant by considering ADLs, physical function, self-reported exhaustion, and symptomatic burden reported by the participant.

#### Delirium screening and assessment

Delirium will be diagnosed by the investigating geriatrician as per the Diagnostic and Statistical Manual of Mental Disorders 5 [50]. Participants will first be screened for evidence of delirium at each visit using the Single Question in Delirium “Do you think this patient has been more confused lately?” [51]. The investigating geriatrician will review notes and ask staff caring for the participant, family members, and the participant themselves. The participant themselves will be specifically asked “Has anything strange been happening?”, such as experiencing hallucinations. Where possibility of delirium is raised upon screening or during other assessments, the investigating geriatrician will perform further assessments to formally diagnose delirium by testing attention by months of the year backwards [52], consciousness by the Modified Richmond Agitation and Sedation Scale [53], and cognition by the Abbreviated Mental Test Score (Supplementary File 2).

#### Fluid balance assessment

Fluid balance will be assessed and recorded during all visits during hospitalisation. Fluid balance will be assessed by clinical assessment, review of input/ output charts, and BIA measurements. Clinical assessment by the investigating geriatrician will include review of skin turgor, mucus membranes, oedema, Jugular Venous Pressure level, trends in observations e.g. blood pressure, and patient presentation. The overall fluid status will be recorded as hypovolaemic, euvolaemic, or hypervolaemic for the participant overall. However, if unilateral oedema is present in a single limb this will be recorded. BIA measurements of TBW, ECW, ICW, and third space water will be recorded separately and assessed against all other available information of fluid balance.

### Other outcome data

Further routinely collected data that will be recorded will include (as applicable) the operation performed, peri-operative blood loss, type of post-operative analgesia (patient-controlled analgesia or epidural), postoperative complications, length of stay, discharge destination, other hospital admissions within the 3 month follow-up period, histological diagnosis (cancer vs. not), and 1 year mortality.

### Participant feedback

There is no standardised assessment tool for measuring test acceptability. However, a multi-faceted construct of acceptability has been proposed, which reflects the extent to which people receiving a healthcare investigation or intervention consider it to be appropriate. This construct consists of the affective attitude of the individual, procedure burden, individual ethicality (individual value system), intervention coherence (participant understanding), opportunity costs, perceived effectiveness, and self-efficacy (confidence that they can perform the necessary behaviour) [54]. Considering this construct, we have devised a questionnaire that assesses each of these aspects separately for muscle quantity (for both ultrasound and BIA), handgrip strength, and gait speed testing (Supplementary file 2). These four aspects have been chosen as these will be measured most frequently for all participants in this study and have potential for direct translation into clinical practice. This will be administered to all participants at their final visit.

### Venepuncture (optional)

Blood samples will be taken using the BD vacutainer Safety-Lok™ system in sterile vacutainers without additives (BD biosciences). Samples will normally be taken peripherally but may be taken centrally or via arterial lines if these are in place as part of routine clinical care. Samples will be taken at first visit, and where possible, within 48 h of surgery in the surgical cohorts. Blood samples will be centrifuged within 30 min to 1 hour of collection, within the University of Birmingham Research Laboratories, within QEHB. Serum and plasma samples will be removed using calibrated pipettes and stored at -80 °C prior to further analysis. Serum and/or plasma concentration levels of high sensitivity C-Reactive Protein (hsCRP), Dehydroepiandrosterone sulfate (DHEA-s), cortisol, 25-OH vitamin D, Interleukin 6 (IL-6), Tumour Necrosis Factor Alpha (TNF-α), and Insulin-like Growth Factor (IGF-1) will be measured using Enzyme-Linked Immunosorbent Assays or other appropriate tests. Further additional biomarkers may be tested as appropriate. Remaining serum and/or plasma samples will be stored for use in future ethically approved research within the University of Birmingham Research Laboratories.

### Fitbit inspire physical activity quantification (optional)

The Fitbit Inspire will be applied to the non-dominant wrist during hospitalisation where this is agreed by the participant or consultee. This will record activity statistics during hospitalisation including number of steps taken, distance travelled, and sedentary time. Summary statistics will be recorded for up until 30 days after hospitalisation. Participants will be advised to wear the monitor all the time. They will be supplied with a charger and advised to charge the device every 5 days when at rest, such as at night time. Position changes (e.g. sit to stand) will not be specifically recorded with this device.

### Statistical analysis

#### Power calculation

The sample size for this study has been calculated by considering estimates of the precision of outcomes; 80% power and 5% significance level have been used in calculating this sample size. Allowing for 25% loss to follow-up from a sample size of 56, based on a paired t-test, the following clinically important changes may be detected with a sample size of 45 in each group (all changes are powered to be bidirectional and may be identified at multiple timeframes):

- Change of 6 for t-score derived from physical function measured by PROMIS (Mean 50, SD = 10) – from baseline to 1 week and/or from baseline to 13 weeks. This is validated from previous studies [39].
- Change of 0.66 cm in BATT (Mean 3.6 cm, SD = 1.1 cm) – from baseline to 1 week and/or from baseline to 13 weeks. This is consistent with clinical change detected in our pilot study (mean loss of 0.76 cm) [24].
- Change of 0.6 in skeletal muscle mass index measured using BIA (Mean 8.5, SD = 1) – from baseline to 1 week and/or from baseline to 13 weeks. This is consistent with change detected in previous studies, consistent with acute sarcopenia [28].
- Change of 6 kg in handgrip strength (Mean 23, SD = 10) – from baseline to 1 week and/or from baseline to 13 weeks. This is validated from previous studies [37].

### Data analysis

Data analysis will be conducted using IBM SPSS® Version 22. Results for each cohort will be analysed separately, although secondary data analysis will be conducted on all groups together. Interim analysis is planned with involvement from a patient and public involvement panel. Outcomes will be summarised at baseline, 7 days postoperatively, and 13 weeks postoperatively. The data analysis of the primary research question will include the following models:

- Unadjusted model with PROMIS at 13 weeks as the outcome of interest, and the secondary outcome as the covariate of interest (i.e. change in BATT, handgrip strength and/or gait speed from baseline to 7 days).
- Adjusted model with PROMIS at 13 weeks as the outcome of interest, and the secondary outcome as the covariate of interest (i.e. change in BATT, handgrip strength and/or gait speed from baseline to 7 days), with adjustment for the baseline PROMIS score, and patient demographics (e.g. age, gender).
- Model with PROMIS at 13 weeks as the outcome of interest, with adjustment for baseline PROMIS, and all secondary outcomes as covariates to establish the strength of association between those secondary outcomes and the primary outcome. This model will be used to establish which of the secondary outcomes (i.e. change in BATT, handgrip strength and/or gait speed from baseline to 7 days) is most strongly associated with change in PROMIS at 13 weeks.

Change in PROMIS rather than change in SPPB, which can be considered an objective measure of physical function, has been selected as our primary outcome for two reasons. Firstly, our patient and public involvement panel considered their own perception of their physical function to be most important. Although perception of function may differ from objective function, how function is perceived for them as individuals was considered more important. Secondly, it will only be possible to obtain true pre-hospitalisation measures of SPPB for the elective cohort. PROMIS provides a method of evaluating physical function prior to admission in the emergency cohorts. Change in SPPB will be evaluated as a secondary outcome in the elective cohort.

### Acute sarcopenia

Sarcopenia will be defined as per EWGSOP2 as handgrip strength below 16 kg in women or below 27 kg in men [2, 55], in combination with low muscle quantity or quality. Cut-offs for low muscle quantity and quality will be evaluated comparing the cohort against reference data in healthy young adults; cut-offs for BATT of 3.85 cm in women and 5.44 cm in men have been proposed [11]. Severe sarcopenia will be defined as additional presence of low physical performance; gait speed 0.8 m/s or less [56] or SPPB of 8 or less [57]. Acute sarcopenia will be defined as incident sarcopenia compared to baseline measurements at recruitment. The prevalence of sarcopenia will be calculated at each visit.

### Patient and public involvement

Older adults have been involved in the design and development of this research. We will host further discussion groups when analysing the results (interim and final). This will be particularly valuable when determining the significance of unexpected results. The interim meeting will be of potential value in assessing if any protocol amendments are necessary. The third discussion group will also be used to co-produce the study report. The findings of this research will be disseminated to all participants and their advocates through a written summary. The participants who are enrolled in this study itself will be the best placed to assess and comment on the acceptability of the procedures used during this study. Within the study design itself, we have devised a questionnaire to derive a multi-faceted acceptability score for assessment of muscle quantity using ultrasound, handgrip strength, and walking speed. We consider that formally interviewing participants could lead to unnecessary burden, given the time they will have already dedicated to the study itself. However, any informal feedback given will be recorded to guide further research and healthcare policy. The final formal meeting that is planned with our discussion group will encompass a full evaluation of the importance of the results of this study and will be used to co-produce any protocols for future research, as well as recommendations for healthcare policy.

### Trial registration

This study was registered on ClinicalTrials.gov (identifier: NCT03858192) on 28th February 2019.

## Discussion

This study will fully characterise changes in muscle quantity and function in a clinical setting and will provide invaluable information to researchers, clinicians, and patients. The results of this study have potential to lead on directly to further interventional studies to counteract these changes, with particular focus on identified mechanistic associations and clinical factors to guide risk stratification. The results may also lead to direct changes in clinical practice, including the embedding of our research tools into clinical practice, and changes in policy, such as promoting early mobilisation. Providing patients and members of the public with increased knowledge on their risk of declines in muscle quantity and function, and what this is likely to mean for them, can help to empower them in their own decision making and engagement with treatment and therapy.

We recognise that there are a number of limitations of our study. Firstly, this is a single site study, and therefore, the results may not be generalisable to the wider population. Secondly, the cohorts we have included are disease-specific. However, we consider that our results will provide proof of concept, which can be used to guide further research to increase understanding in other disease populations. As described, our study has been powered to detect within group differences in the minimally clinical important differences as derived from other studies. However, this has not been powered separately for gender and other covariates (e.g. cancer vs. not), which may affect measurements. Interim analysis has been planned and our patient and public involvement panel will be involved in the interpretation of these results. At this stage, we will review the overall progress of the study and consider if protocol amendments may be necessary.

There is some evidence that position can affect measurement of muscle quantity by ultrasound and BIA [58, 59]. However, the results will be compared to a reference group of young healthy individuals taken in the same position as we have described, using the same technique [11]. The position described is one that we can consider to be feasible for measurements in a variety of different clinical environments, whilst ensuring the quadriceps are relaxed. Particular care will be taken to standardise the position of each measure for each participant across separate visits. Nevertheless, we acknowledge that due to measurements being taken in different clinical environment, there may be small uncontrollable differences in position across visits.

Venepuncture and physical activity recording have both been included as optional aspects of the study, and it is not known what percentage of participants will agree to these. However, venepuncture was previously included as an optional aspect within our pilot study and all participants were in agreement with this [24]. We recognise that there will be limitations of physical activity measurements recorded through the Fitbit Inspire. These devices will be unable to specifically measure change in position (e.g. sit to stand). Previous studies using raw accelerometer data have shown a floor effect when measuring physical activity in frail, sedentary older adults [60]. However, physical activity measures using Fitbits have also shown to correlate well with raw accelerometer data in studies involving older adults [61]. The Fitbit Inspire is considered to be an acceptable device for older adults due to its simple wristwatch-like design, and their low cost means that they are potentially utilisable in clinical practice. Within our study, we will assess the feasibility of using these devices and assess the validity of data recorded as covariates and predictors of change in muscle parameters and physical function.

Despite these limitations, we consider the recruitment of a complex heterogeneous population to be a strength of this study. Frail older adults are frequently under-represented in research studies. It is not possible to be certain that changes seen in young healthy adults are concordant with changes in older adults. Determining the mechanisms involved in the development of acute sarcopenia will enable risk stratification, and targeted interventions to prevent or even reverse the effects.

## Supplementary information

**Additional file 1.**

**Additional file 2.**

## Data Availability

Anonymised data will be available from the corresponding author upon reasonable request.

## Abbreviations

ADLs: Activities of Daily Living
CFS: Clinical Frailty Scale
cm: Centimetres
DHEA-s: Dehydroepiandrosterone sulfate
FI: Frailty Index
hsCRP: High sensitivity C-Reactive Protein
IGF-1: Insulin-like Growth Factor 1
IL-6: Interleukin 6
kg: Kilograms
L3: Third Lumbar vertebra
MNA: Mini-Nutritional Assessment
PROMIS: Patient Reported Outcome Measures Information System
SD: Standard Deviation
SPPB: Short Physical Performance Battery
TNF-α: Tumour Necrosis Factor Alpha
TPA: Total Psoas Area
