# Supplementary material for: Protocol for understanding acute sarcopenia: a cohort study to characterise changes in muscle quantity and physical function in older adults following hospitalisation
Source: BMC Geriatr. 2020 Jul 10;20:239. doi: 10.1186/s12877-020-01626-4 (PMC7350619; doi:10.1186/s12877-020-01626-4)
Supplement: Supplementary file 1 — Additional file 1. [file 12877_2020_1626_MOESM1_ESM.docx]

**Criteria used to form Frailty Index**

| Deficit | Definition |
| --- | --- |
| Activity limitation | Positive Fried physical activity score |
| Anaemia and haematinic deficiency | As per local reference ranges (female Hb<115, male Hb<135) or on medication for haematinic deficiency |
| Arthritis | Patient reported (includes osteoarthritis and inflammatory arthritis) |
| Atrial fibrillation | Any history – paroxysmal, temporary, or permanent |
| Cerebrovascular disease | Vascular dementia or stroke disease |
| Chronic kidney disease | eGFR <60 |
| Diabetes mellitus | Known history/ confirmed diagnosis |
| Dizziness | Patient reported |
| Dyspnoea | Patient reported |
| Falls | Two or more over previous year |
| Foot problems | Patient reported |
| Fragility fracture | Previous history |
| Hearing impairment | Need for hearing aids |
| Heart failure | Known history/ confirmed diagnosis |
| Heart valve disease | Known history |
| Housebound | Lawton instrumental ADLs |
| Hypertension | On treatment or recorded |
| Presyncope/ syncope | Patient reported (altered from “hypotension” in original eFI) |
| Ischaemic heart disease | Known history |
| Memory and cognitive problems | Any cognitive spectrum disorder including mild cognitive impairment, delirium, and dementia |
| Osteoporosis | On treatment or known history |
| Parkinsonism and tremor | Includes tremor of any cause – known history or on treatment |
| Peptic ulcer | Known history |
| Peripheral vascular disease | Known history |
| Polypharmacy | ≥5 prescribed medications |
| Requirement for care | Formal carers |
| Respiratory disease | Any history of chronic disease e.g. asthma, COPD |
| Skin ulcer | Present history as per Mini Nutritional Assessment (MNA) – patient reported |
| Sleep disturbance | Patient reported |
| Social vulnerability | Lives alone |
| Thyroid disease | Known history |
| Urinary or faecal incontinence | Katz ADLs |
| Urinary system disease | Known history |
| Visual impairment | Wears glasses/ visual aids or on treatment for eye condition(s) |
| Weight loss and anorexia | Fried weight loss OR MNA weight loss OR MNA intake decline |

**Delirium assessment**

| **a** | A disturbance in;  i) Attention- reduced ability to direct, focus, sustain, and shift attention FROM; *20-1, MOYB, observation* | | | **Yes** | No | ? |
| --- | --- | --- | --- | --- | --- | --- |
|  | ii) Awareness (reduced orientation to the environment) FROM; *mRASS not 0, observation* | | | **Yes** | No | ? |
| **b** | The disturbance;  i) Develops over a short period of time (usually hours to a few days) | | | **Yes** | No | ? |
|  | Ii) Represents a change from baseline attention & awareness and iii) tends to fluctuate in severity during the course of the day FROM: *History* | | | **Yes** | No | ? |
| **c** | An additional disturbance in cognition (e.g. memory deficit, disorientation, language, visuospatial ability, or perception). FROM; *Describe a pen, describe the morning, AMTS questions, observation* | | | **Yes** | No | ? |
| **d** | Exclusions- The disturbance in criteria A and C are;  i) Better explained by another pre-existing, established, or evolving neurocognitive disorder, or ii) Occur in the context of a severely reduced level of arousal such as coma. FROM; *mRASS not -4, -5* | | | Yes | **No** | ? |
| **e** | There is evidence from the history, physical examination or laboratory findings that the disturbance is a direct physiological consequence of another medical condition, substance intoxication or withdrawal, or exposure to a toxin, or is due to multiple aetiologies. FROM*; Notes (likely to be yes as in hospital)* | | | **Yes** | No |  |
|  | **Probable Delirium Diagnosis – all items a,b,c and e ‘yes’, plus d ‘no’** | **Yes** | **No** | | |  |
|  | Possible delirium diagnosis – if any ‘?’ or e ‘no’ | Yes | No | | |  |
