# Supplementary material for: Protocol for understanding acute sarcopenia: a cohort study to characterise changes in muscle quantity and physical function in older adults following hospitalisation
Source: BMC Geriatr. 2020 Jul 10;20:239. doi: 10.1186/s12877-020-01626-4 (PMC7350619; doi:10.1186/s12877-020-01626-4)

|  | Strongly disagree (1) | Disagree  (2) | Neither  (3) | Agree  (4) | Strongly agree (5) |
| --- | --- | --- | --- | --- | --- |
| I enjoyed participating in this test | ⃝ | ⃝ | ⃝ | ⃝ | ⃝ |
| Minimal effort was required to participate in this test | ⃝ | ⃝ | ⃝ | ⃝ | ⃝ |
| This test was unobtrusive | ⃝ | ⃝ | ⃝ | ⃝ | ⃝ |
| I understand how this test works and its importance | ⃝ | ⃝ | ⃝ | ⃝ | ⃝ |
| This test was not time-consuming | ⃝ | ⃝ | ⃝ | ⃝ | ⃝ |
| This test is likely to have a positive impact on patients | ⃝ | ⃝ | ⃝ | ⃝ | ⃝ |
| I felt confident that I could complete this test | ⃝ | ⃝ | ⃝ | ⃝ | ⃝ |


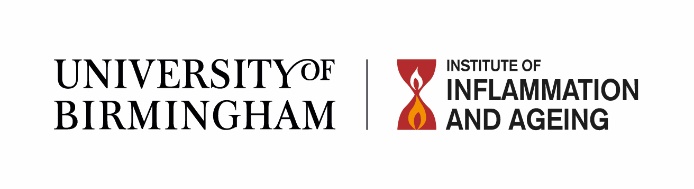
Considering the testing of **handgrip strength**, please rate your agreement with the following:


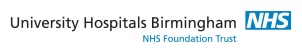
Considering the testing of **walking speed**, please rate your agreement with the following statements:

|  | Strongly disagree (1) | Disagree  (2) | Neither  (3) | Agree  (4) | Strongly agree (5) |
| --- | --- | --- | --- | --- | --- |
| I enjoyed participating in this test | ⃝ | ⃝ | ⃝ | ⃝ | ⃝ |
| Minimal effort was required to participate in this test | ⃝ | ⃝ | ⃝ | ⃝ | ⃝ |
| This test was unobtrusive | ⃝ | ⃝ | ⃝ | ⃝ | ⃝ |
| I understand how this test works and its importance | ⃝ | ⃝ | ⃝ | ⃝ | ⃝ |
| This test was not time-consuming | ⃝ | ⃝ | ⃝ | ⃝ | ⃝ |
| This test is likely to have a positive impact on patients | ⃝ | ⃝ | ⃝ | ⃝ | ⃝ |
| I felt confident that I could complete this test | ⃝ | ⃝ | ⃝ | ⃝ | ⃝ |


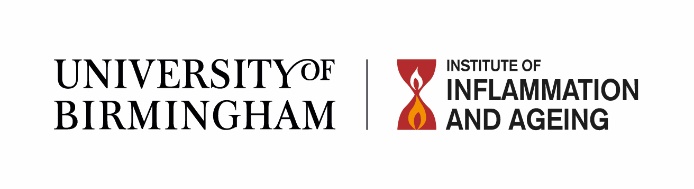
Considering the testing of **muscle mass using ultrasound**, please rate your agreement:

|  | Strongly disagree (1) | Disagree  (2) | Neither  (3) | Agree  (4) | Strongly agree (5) |
| --- | --- | --- | --- | --- | --- |
| I enjoyed participating in this test | ⃝ | ⃝ | ⃝ | ⃝ | ⃝ |
| Minimal effort was required to participate in this test | ⃝ | ⃝ | ⃝ | ⃝ | ⃝ |
| This test was unobtrusive | ⃝ | ⃝ | ⃝ | ⃝ | ⃝ |
| I understand how this test works and its importance | ⃝ | ⃝ | ⃝ | ⃝ | ⃝ |
| This test was not time-consuming | ⃝ | ⃝ | ⃝ | ⃝ | ⃝ |
| This test is likely to have a positive impact on patients | ⃝ | ⃝ | ⃝ | ⃝ | ⃝ |
| I felt confident that I could complete this test | ⃝ | ⃝ | ⃝ | ⃝ | ⃝ |

Considering **bioelectrical impedance analysis**, please rate your agreement with the following:

|  | Strongly disagree (1) | Disagree  (2) | Neither  (3) | Agree  (4) | Strongly agree (5) |
| --- | --- | --- | --- | --- | --- |
| I enjoyed participating in this test | ⃝ | ⃝ | ⃝ | ⃝ | ⃝ |
| Minimal effort was required to participate in this test | ⃝ | ⃝ | ⃝ | ⃝ | ⃝ |
| This test was unobtrusive | ⃝ | ⃝ | ⃝ | ⃝ | ⃝ |
| I understand how this test works and its importance | ⃝ | ⃝ | ⃝ | ⃝ | ⃝ |
| This test was not time-consuming | ⃝ | ⃝ | ⃝ | ⃝ | ⃝ |
| This test is likely to have a positive impact on patients | ⃝ | ⃝ | ⃝ | ⃝ | ⃝ |
| I felt confident that I could complete this test | ⃝ | ⃝ | ⃝ | ⃝ | ⃝ |


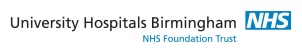


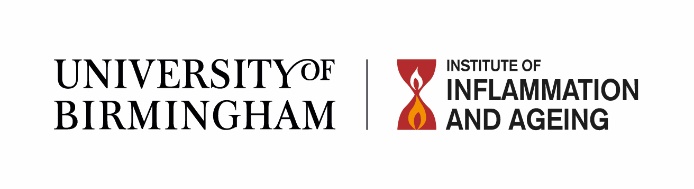
Any further comments related to this study?


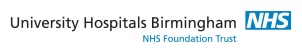

Supplement: Supplementary file 2 — Additional file 2. [file 12877_2020_1626_MOESM2_ESM.docx]
